# Development and psychometric validation of a self-administered questionnaire assessing the acceptance of influenza vaccination: the Vaccinees' Perception of Injection (VAPI^©^) questionnaire

**DOI:** 10.1186/1477-7525-7-21

**Published:** 2009-03-04

**Authors:** Catherine Chevat, Muriel Viala-Danten, Carla Dias-Barbosa, Van Hung Nguyen

**Affiliations:** 1 Sanofi Pasteur, 2, avenue du Pont Pasteur, 69007 Lyon, France; 2 Mapi Values, 27, rue de la Villette, 69003 Lyon, France

## Abstract

**Background:**

Influenza is among the most common infectious diseases. The main protection against influenza is vaccination. A self-administered questionnaire was developed and validated for use in clinical trials to assess subjects' perception and acceptance of influenza vaccination and its subsequent injection site reactions (ISR).

**Methods:**

The VAPI questionnaire was developed based on interviews with vaccinees. The initial version was administered to subjects in international clinical trials comparing intradermal with intramuscular influenza vaccination. Item reduction and scale construction were carried out using principal component and multitrait analyses (n = 549). Psychometric validation of the final version was conducted per country (n = 5,543) and included construct and clinical validity and internal consistency reliability. All subjects gave their written informed consent before being interviewed or included in the clinical studies.

**Results:**

The final questionnaire comprised 4 dimensions ("bother from ISR"; "arm movement"; "sleep"; "acceptability") grouping 16 items, and 5 individual items (anxiety before vaccination; bother from pain during vaccination; satisfaction with injection system; willingness to be vaccinated next year; anxiety about vaccination next year). Construct validity was confirmed for all scales in most of the countries. Internal consistency reliability was good for all versions (Cronbach's alpha ranging from 0.68 to 0.94), as was clinical validity: scores were positively correlated with the severity of ISR and pain.

**Conclusion:**

The VAPI questionnaire is a valid and reliable tool, assessing the acceptance of vaccine injection and reactions following vaccination.

**Trial registration:**

NCT00258934, NCT00383526, NCT00383539.

## Background

Influenza is among the most common infectious diseases worldwide and is caused by influenza viruses (A, B, C) [1]. The disease occurs in all age groups with the highest infection rate described in children [2-4]. The highest rates of morbidity and mortality are reported among people over 65 years of age and children under 23 months [4,5]. Age, chronic underlying diseases and immunosuppressive medical conditions increase the likelihood of complications in case of influenza and can lead to death especially among people aged 65 years or more [6]. Because of sick leave, family disturbance, loss of productivity and health care costs, the socio-economic and public health impact of the disease is significant [7,8]. Annual vaccination is currently recommended by many public health authorities for individuals in high risk groups and those who are particularly exposed to the disease [9,10]. Despite recommendations and the proven effectiveness of influenza vaccines in reducing both the health and economic burden of the disease, vaccination remains underused [11-16].

For more than 60 years, intramuscular (IM) vaccination of inactivated influenza vaccine has formed the cornerstone of seasonal influenza prevention. The intradermal (ID) route of administration is an interesting alternative in influenza as well as in other diseases (e.g. hepatitis B, rabies), with demonstrated immunogenicity in the elderly and in younger adults [17-22]. In the elderly, the ID route has shown a higher Haemagglutination Inhibition (HI) antibody immunogenic response than the IM route with the same quantity of viral antigens [23]. However, as ID vaccination involves the injection of these antigens just below the skin surface, injection site reactions (ISR) such as erythema, prutitus, pain, swelling, and other local reactions are more frequently visible and reported with the ID route than with the IM route [21].

Clinical trials were conducted to compare ID influenza vaccination (administered using a newly developed micro-injection system) with conventional IM influenza vaccination. In order to complement traditional safety information, we wanted to assess the vaccinees' perspective regarding injection and local reactions. Although symptoms related to systemic or local reactions induced by influenza vaccination have often been reported using patients' questionnaires [24-26], these questionnaires do not investigate the acceptance of vaccine injection and its subsequent local reactions. Acceptance is the consent to the bother resulting from local reactions, and their impact on physical functioning, emotional well-being and social activities.

We therefore developed the VAccinees' Perception of Injection questionnaire (VAPI) and included it as an exploratory tool in three international clinical trials comparing ID and IM vaccines. In addition to evaluating the level of inconvenience ("bother") caused by ISR, their impact on sleep and on arm movements, and the subjects' ability to tolerate ISR ("acceptability of ISR"), the questionnaire assesses how anxious subjects are before vaccination and (after vaccination) how willing they are to be vaccinated the following year, as well as their overall satisfaction with the micro-injection system.

We report the development, finalisation, scoring and psychometric validation steps of the VAPI questionnaire.

## Methods

### Development of the questionnaire

Firstly, the literature was reviewed to identify instruments that assess the importance and the acceptability of ISR to subjects and the impact of these on subjects' daily life, as well as the overall acceptance of the administration route. Investigations to identify scales for pain assessment during injection were also conducted.

Secondly, interviews were conducted in the United States (US), Germany and Switzerland (French-speaking) to explore the perceptions of influenza vaccination, injection site pain and other ISR, the impact of reactions on daily life and barriers related to the vaccination. As we were interested in the subjects' perception and the impact of ISR, and not in a self-reported evaluation of the severity of local reactions, these interviews were conducted 21 days after vaccination. This time period was long enough for the majority of ISR to have remitted, but short enough for subjects to remember their experience with ISR. Subjects from Germany and Switzerland were participating in a phase II clinical trial and had received either ID or IM influenza vaccination. Subjects from the US were recruited via their general practitioners, and had received IM influenza vaccination. Interviewees were recruited among adult subjects, male or female, who had received either ID or IM influenza vaccination, and had reported at least one injection site reaction during the week following injection. Prior to the interview, all the included patients had to sign an informed consent form. Interview guides were developed for in-depth face-to-face and semi-structured interviews. These guides were approved by ethics committees in the respective countries.

All interviews were audio-taped. Subjects' own words were transcribed in the language in which the interviews were conducted, by a native speaker of that language. Qualitative analysis of transcripts consisted in classifying subjects' quotes into domains and sub-domains. Comments pertaining to the research questions were highlighted. A conceptual framework with the main domains identified was then developed, providing an understanding of the relationships between various aspects of the acceptance of the vaccination.

Interviewees were also asked to give their preference between three different pain rating scales: a visual analogue scale (VAS), a 6-point Likert verbal rating scale (VRS), and a 10-point numerical rating scale (NRS). Based on the analysis of the interview transcripts, US English, German and French versions of a questionnaire were developed during a two-day meeting with a team that included native speakers of each target language.

The content and the format of the questionnaire were subsequently comprehension tested during a second set of interviews with US, German and French subjects selected according to the same criteria as described above, and with additional subjects recruited among older adults (> 60 years) from an ID influenza vaccine clinical trial conducted in Australia. The respondents were asked to complete the questionnaire and answer questions about its content, structure, item relevance and ease of comprehension.

### Finalisation and validation of the questionnaire

#### Study design and populations

The questionnaire was used during three multicentre randomised controlled clinical trials that aimed to assess the HI antibody immunogenicity 21 days after influenza vaccination using the ID new micro-injection system or the conventional IM route (trial registration codes: NCT00258934, NCT00383526, NCT00383539). Clinical trials included healthy adults aged 18–60 years or seniors aged over 60 years. All subjects gave their written informed consent before being included in the clinical studies and received ID or IM vaccine according to the randomisation at inclusion. The initial version of the VAPI questionnaire was translated and culturally adapted into Belgian Dutch, UK English, Spanish and Italian. It was completed 21 days after vaccination by subjects from France, Belgium, Germany, Italy, the United Kingdom and Spain. Data collected during the three clinical trials were pooled to finalise and validate the questionnaire.

Subjects whose VAPI questionnaires were completed with less than 50% of missing items were included in the overall analysis. As the reference language for questionnaire development was English, questionnaire finalisation analysis (i.e. item reduction and scale definition) was therefore performed in the "UK finalisation population" set constituted of two-thirds of the UK clinical trial subjects. The scale structure was then validated in the remaining one-third of the "UK validation population", to assess the robustness of the analyses in an independent sample, as well as in the other 5 countries. The reliability and validity of the final VAPI questionnaire were assessed per country and in the pooled population from all six countries.

#### Statistical analyses and psychometric properties

Data collected with the VAPI are non-normal as subjects' answers are on 5-point ordinal scales, hence non-parametric methods were used. In order to finalise the structure of the VAPI questionnaire, a factorial approach was chosen in order to analyse the questionnaire in its entirety. Principal component analysis (PCA) using Varimax rotation was performed and interpreted in the light of the conceptual framework developed during the qualitative step of this work. The Kaiser-Guttman criterion was used to retain factors (i.e. Eigen values greater than one) [27], and the content of each item grouped onto a factor was analysed before defining the factor as a single concept. The final structure of the VAPI questionnaire was confirmed using Multitrait Analysis (MA) based on item-scale Spearman correlations [28]. The correlation between each item and its own scale was considered satisfactory if it achieved ≥ 0.40 (item convergent validity). Item discriminant validity requires that each of the items shares a higher correlation with its own dimension than with other dimensions [29]. Floor and ceiling effects were determined to check that there were no issues related to a high percentage of patients having the lowest or the highest possible score, respectively. Questionnaire scale-scale correlations were determined by calculating Spearman coefficients. Clinical validity, defined by Chassany *et al *[30] as the ability of an instrument to discriminate between groups of patients whose health status differs, was assessed by the description and comparison of the questionnaire scores according to ISR severity as reported in the clinical trial case report forms using Mann-Whitney-Wilcoxon (when comparing two groups of patients) and Kruskal-Wallis (when comparing three or more groups of patients) non-parametric tests. VAPI scores were also described according to subjects' age and severity of systemic reactions. Internal consistency reliability of the questionnaire was assessed by determining the Cronbach's alpha coefficient [31]: a value of 0.70 or above was considered satisfactory for group comparisons [32]. Spearman correlation coefficients were calculated between items related to each ISR and the maximum severity of the corresponding reaction.

The threshold for statistical significance was fixed at 5%. All data processing and analyses were performed using SAS software (Statistical Analysis System, Version 9).

## Results

### Development of the questionnaire

**Figure 1:**
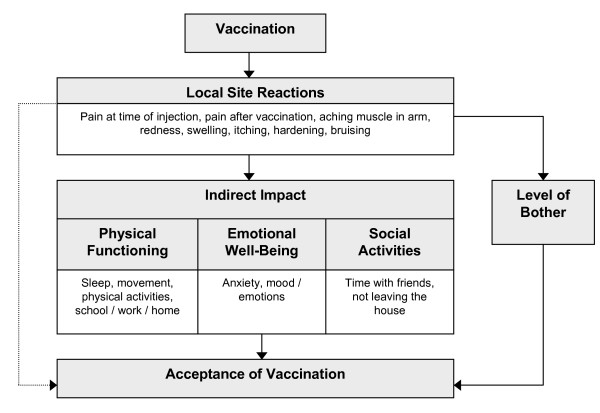
**Conceptual framework**.

Thirty-three subjects (aged 18–74 years old) from the US (n = 10), Germany (n = 15), and Switzerland (n = 8) were interviewed face-to-face. Interviews were analysed and organised within a conceptual framework composed of different domains composing "acceptance of vaccination" (Figure 1). Based on this framework and using subjects' own words, items were simultaneously generated in US English, German and French.

The questionnaire was comprehension-tested with 23 subjects. Comments made by interviewees during comprehension testing by subjects aged 18–60 (5 US, 5 German and 5 French subjects), and by subjects aged > 60 year old (8 Australian subjects vaccinated either intradermally, n = 4; or intramuscularly, n = 4), were used to modify the questionnaire to improve clarity. The 6-point VRS scale was preferred over the 10-point NRS and the VAS by subjects.

The test version of the VAPI questionnaire contained 44 items divided into 6 main domains covering the level of bother caused by ISR (9 items), impact of reactions on subjects' emotional well-being (8 items), physical functioning (18 items), social activities (2 items), and on subjects' acceptance of vaccination (4 items) and intention to be vaccinated again (3 items). The questionnaire was self-administered and was as well adapted to adults as to the elderly.

### Finalisation and psychometric validation of the questionnaire

#### Description of the population

Socio-demographic and clinical characteristics of the subjects are summarised in Table 1. Six thousand and ninety-two subjects completed more than 50% of the questionnaire. Overall mean age was 57 years, ranging from 24 years in Spain to 70 years in Italy. There were more women than men in all countries except in Italy. Most subjects (99% of the total population) were Caucasian. The majority of subjects (63%) had a history of influenza vaccination, although the proportion ranged widely from 28% in Germany to 92% in Italy.

**Table 1:** Socio-demographic and clinical characteristics of the population at baseline

	**Belgium (n = 1,451)**	**France (n = 3,243)**	**Spain (n = 201)**	**The United Kingdom (n = 864)**	**Germany (n = 109)**	**Italy (n = 224)**	**Total (n = 6,092)**
**Age: Mean (STD)**	55.43 (17.09)	62.71 (15.28)	24.08 (5.76)	46.97 (10.52)	40.08 (10.96)	69.80 (6.78)	**57.33 (17.13)**

**Gender: Female %**	57	56	70	57	58	43	**56**

**Ethnic origin:**							
**Asian: N(%)**	2 (<1)	4 (<1)	0 (0)	10 (1)	2 (2)	0 (0)	**18 (<1)**
**Black: N(%)**	2 (<1)	13 (<1)	2 (1)	6 (1)	0 (0)	0 (0)	**23 (<1)**
**Caucasian: N(%)**	1,443 (99)	3,218 (99)	197 (98)	844 (98)	105 (96)	224 (100)	**6,031 (99)**

**Previous vaccination:**							
**Yes: N(%)**	896 (62)	2,106 (65)	80 (40)	535 (62)	31 (28)	207 (92)	**3,855 (63)**
**Unknown: N(%)**	18 (1)	35 (1)	5 (2)	8 (1)	5 (5)	0 (0)	**71 (1)**

#### Return rate and quality of completion of the questionnaire

Return rates of the questionnaires ranged from 73% (Germany) to 99% (Belgium and France). Among the 6,126 questionnaires returned (overall return rate of 95%), 5,121 (83.59%) had no missing data, and 6,092 (99%) had less than 50% missing data. The mean percentage of missing items per subject was 2.26%, with the lowest percentage reported for Spain (0.37%) and the highest for Italy (3.28%).

#### Finalisation and validation of the questionnaire scale structure

##### Item reduction process and finalisation

The purpose of this step was to explore the links between the questionnaire items to define the number of dimensions and to establish a consistent scoring algorithm. The "UK finalisation population" set was used, corresponding to two thirds (n = 549) of the subjects from this country, who completed at least 50% of the items of the questionnaire. A series of PCA with Varimax rotation and MA resulted in the deletion of 23 items; 16 items did not display variability (more than 90% of the subjects ticked the same response-choice), the content of one item was inconsistent with that of the other items in its dimension, the response-choices of one item were not interpretable, 2 items were distributed on several dimensions, 2 items were redundant with items of their own dimension, and the wording of one item was found to be confusing by subjects.

The last PCA was performed with 521 subjects (complete questionnaire) from the "UK finalisation population" set and yielded four factors with eigenvalues of greater than one, accounting for 69% of the total variance (Table 2): the first factor consisted of 6 items (#3 to 8) related to subjects' bother from the ISR following vaccination, and thus was labelled as the "bother from ISR" dimension, the second factor consisted of 4 items (#11, 12 and 25, 26) related to the impact of reactions on carrying and lifting, and was labelled "arm movement"; the third factor contained 4 items (#13, 14 and 27, 28) concerning sleep, and was referred to as the "sleep" dimension; the fourth factor consisted of 2 items (#38, 39) related to the tolerability of local reactions for subjects, and was defined as the "acceptability" dimension. Five items were not included in any of these dimensions and were maintained as individual items. They measured subjects' bother due to the pain during vaccination (item 2), their anxiety before vaccination (item 1) and about the next vaccination (item 42), their willingness to be vaccinated the following year (item 43), and their overall satisfaction with the delivery system (item 40). Dimensions, individual items and item content of the VAPI questionnaire are presented in Table 3.

**Table 2:** Principal Component Analysis after item reduction on the "UK Finalisation Population" set (N = 521)

**# item and item content**	**Factor 1**	**Factor 2**	**Factor 3**	**Factor 4**
**5. Bothered by swelling?**	0.804	0.111	0.180	0.189
**7. Bothered by hardening (a bump)?**	0.797	0.069	0.146	0.102
**4. Bothered by redness?**	0.793	0.054	0.099	0.148
**6. Bothered by itching?**	0.656	0.041	0.013	0.049
**8. Bothered by bruising?**	0.579	0.257	0.126	0.016
**3. Bothered by pain in your arm?**	0.556	0.258	0.309	0.178

**14. Difficulties caused by local reaction(s) when picking up or carrying heavy objects?**	0.118	0.869	0.221	0.027
**28. Difficulties caused by pain when picking up or carrying heavy objects?**	0.094	0.795	0.325	0.075
**13. Difficulties caused by local reaction(s) in moving or lifting arm?**	0.181	0.793	0.195	0.136
**27. Difficulties caused by pain in moving or lifting arm?**	0.177	0.746	0.287	0.211

**26. Affected by pain when changing positions during night?**	0.102	0.261	0.844	0.100
**12. Affected by local reaction(s) when changing positions during night?**	0.199	0.295	0.802	0.074
**25. Bothered by pain when falling asleep?**	0.120	0.209	0.783	0.169
**11. Bothered by local reaction(s) when falling asleep?**	0.207	0.222	0.740	0.077

**39. Acceptable pain?**	0.126	0.234	0.207	0.880
**38. Acceptable local reaction(s)?**	0.305	0.074	0.107	0.869

**Table 3:** Final structure of the VAPI questionnaire

**Dimension**	**Number of items**	**Item Content**
**Bother from ISR**	6	Pain in arm
		Redness at vaccination site
		Swelling at vaccination site
		Itching at vaccination site
		Hardening at vaccination site
		Bruising at vaccination site

**Arm movement**	4	Local reaction(s) and difficulties in moving/lifting arm
		Local reaction(s) and difficulties in picking up/carrying heavy objects
		Pain and difficulties in moving/lifting arm
		Pain and difficulties in picking up/carrying heavy objects

**Sleep**	4	Local reaction(s) and falling asleep?
		Local reaction(s) and changing positions during the night
		Pain and falling asleep
		Pain and changing positions during the night

**Acceptability**	2	Acceptability of local reaction(s)
		Acceptability of pain

**Items analysed separately**	5	Anxiety before receiving vaccination
		Bother due to pain during vaccination
		Satisfaction with injection system (needle, syringe) used for vaccination
		Anxiety about receiving vaccination next year
		Willingness to be vaccinated again next year

The VAPI questionnaire is protected by copyright with all rights reserved to sanofi pasteur. Do not use without permission. However, sanofi pasteur kindly encourages the use of the VAPI questionnaire by clinicians and researchers. For information on, or permission to use the questionnaire, please contact Van Hung Nguyen, sanofi pasteur, 2 Avenue du Pont Pasteur 69007 Lyon, FRANCE. Tel: +33 (0)4 37 37 78 68.

Email: VanHung.Nguyen@sanofipasteur.com

##### Validation of the final structure of the VAPI questionnaire and scoring

All items met the convergent validity criterion for all four dimensions, with the exception of one item (item 8) in France and Germany and two items in Spain (items 6 and 8) in the "bother from ISR" dimension, and one item (item 14) in the "arm movement" dimension in Italy. Regardless of the country, all items met the discriminant validity criterion within their corresponding dimension, except for a single item (item 3) in the "bother from ISR" dimension in Belgium, Spain and Germany, which was more correlated to the "arm movement" dimension for Belgium and Germany and to the "sleep" dimension for Spain.

For each multi-item dimension, a score was determined if at least 50% of the items within its dimension were completed; otherwise, the score was considered as missing. The score of a dimension was calculated as the mean of all items within the dimension. Individual items were analysed separately. Scores ranged from 1 to 5, and questions were phrased in such a way as to ensure that 1 always equated with the most favourable perception of vaccination, and 5 the most unfavourable (e.g. the highest level of bother due to pain and ISR, the greatest difficulty in moving the arm, the highest impact on sleep and the lowest acceptability of ISR or willingness to be vaccinated the following year).

#### Psychometric properties of the final version of the VAPI questionnaire

##### Distribution of the scores

Mean scores of the four dimensions and the five individual items were very low, ranging from 1.08 to 1.31 for the "arm movement" and "bother from ISR" dimensions, and from 1.11 (item 42, regarding the anxiety about next vaccination) to 1.58 (item 43, regarding the willingness to be vaccinated the following year).

The percentage of subjects with the minimum score (i.e. 1) ranged from 43% for the "bother from ISR" dimension to 89% for the "arm movement" dimension for the total population; values ranged from 13% for the "bother from ISR" dimension among the Spanish subjects to 94% for the "arm movement" dimension among the French subjects.

##### Internal consistency reliability

Regardless of the country, Cronbach's alpha values ranged from 0.81 for the "bother from ISR" dimension to 0.90 for the "arm movement" dimension (Table 4), indicating the very good internal consistency of the items constituting a dimension. By country, Cronbach's alpha ranged from 0.73 for the "bother from ISR" dimension in Spain and for the "arm movement" dimension in Italy to 0.94 for the "arm movement" and "sleep" dimensions in Germany. Cronbach's alpha was slightly lower for the "sleep" dimension in Italy (0.68).

**Table 4:** Cronbach's alpha of the multi-item dimensions

	**Belgium (n = 1,383)**	**France (n = 3,084)**	**Spain (n = 198)**	**The United Kingdom (n = 837)**	**Germany (n = 107)**	**Italy (n = 209)**	**Total (n = 5,818)**
**Bother from ISR**	0.81	0.78	0.73	0.83	0.81	0.85	**0.81**
**Arm movement**	0.91	0.91	0.90	0.89	0.94	0.73	**0.90**
**Sleep**	0.91	0.84	0.90	0.88	0.94	0.68	**0.88**
**Acceptability**	0.80	0.88	0.83	0.84	0.81	0.90	**0.85**

ISR, injection site reactions

##### Clinical validity

**Figure 2:**
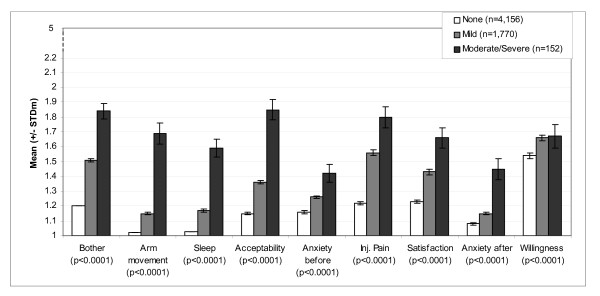
**VAPI scores according to maximum severity of pain reported for 8 days after injection**. Mean scores and standard deviation of the mean (STDm); p = p-value of Kruskal-Wallis test; N = 6,092.

**Figure 3:**
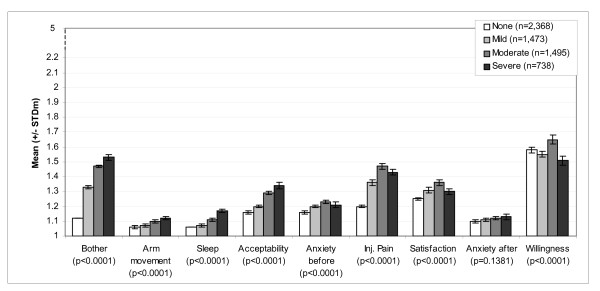
**VAPI scores according to maximum severity of erythema reported for 8 days after injection**. Mean scores and standard deviation of the mean (STDm); p = p-value of Kruskal-Wallis test; N = 6,092.

Scores of dimensions and individual items of the questionnaire are presented according to maximum severity of pain in Figure 2 and maximum severity (size) of erythema in Figure 3, reported over an 8-day period (between D0 and D7). Scores analysed according to the severity of other ISR followed the same pattern (data not shown). For each of the ISR considered (pain, pruritus, erythema, swelling, induration and ecchymosis), mean questionnaire scores increased with increasing severity of the corresponding reaction reported in the case report forms: i.e. scores were highest among subjects who reported the highest level of injection site pain during injection. Differences between groups of severity were statistically significant for all ISR (Kruskal-Wallis p-values < 0.0001). The same pattern was observed with the individual items: milder reactions were associated with lower scores for items 1 ("anxiety before vaccination"), 42 ("anxiety for the next vaccination"), 2 ("bother from pain during vaccination") and 40 ("satisfaction with the injection system"). The link between the severity of reactions and willingness to be vaccinated the following year (item 43) was less clear.

Regarding systemic reactions (fever, headache, malaise, myalgia and shivering), a similar trend was reported, i.e. mean scores increased as the maximum severity of the solicited systemic reaction increased.

When looking at the description of the scores according to subjects' age groups, mean scores were lower in older subjects, with the differences between age groups being statistically significant (p < 0.0001).

##### Correlation between the maximum severity of ISR and the questionnaire scores of the corresponding items

Spearman correlation coefficients between the severity of each reaction reported for 8 days after the injection using the verbal rating scale, and the respective items of the "bother from ISR" dimension of the questionnaire varied from 0.14 to 0.42 (Table 5). A moderate correlation (0.42) was observed between the scores of item regarding the bother of pain at the injection site (item 2) and the pain reported by subjects immediately after the injection.

**Table 5:** Correlation between VAPI item scores and maximum severity of pain and injection site reactions

**Items of the VAPI questionnaire**	**Injection site reaction reported in the case report form**	**Spearman correlation coefficients (n)**
**2. Bothered by pain during the vaccination?**	Pain during injection	0.42 (6,047)
**3. Since your vaccination, bothered by pain in your arm?**	Pain	0.21 (1,917)
**4. Bothered by redness?**	Erythema	0.15 (3,694)
**5. Bothered by swelling?**	Swelling	0.19 (2,272)
**6. Bothered by itching?**	Pruritus	0.18 (1,698)
**7. Bothered by hardening (a bump)?**	Induration	0.16 (2,415)
**8. Bothered by bruising?**	Ecchymosis	0.14 (321)

Spearman correlation coefficients calculated between the VAPI item scores and the maximum severity of pain during injection, and pain and injection site reaction reported for 8 days after injection; N_total _= 6,092

## Discussion

Conventional influenza vaccines are currently administered via the IM route and induce significant and protective HI antibody immune responses in adults and in seniors, although the response is lower in the latter [33]. The ID route has been demonstrated to be a valuable and effective alternative, but visible ISR are frequently reported when the vaccine is injected just below the skin surface.

The vaccinees' perception of injection questionnaire (VAPI) was developed to assess subjects' perception and attitudes concerning vaccination and any ISR that may occur. The VAPI does not provide a self-reported evaluation of the severity of ISR, as many other patient-questionnaires do [24-26], but it investigates subjects' acceptance of influenza vaccination and its subsequent ISR 21 days following injection. Several studies have reported the predictors of the overall acceptance of vaccination, but did not focus on ISR [34-38]. The development of the VAPI followed a rigorous and recommended standard procedure [30], based on in-depth interviews and subsequent comprehension testing with vaccinated adults, to allow the subjects' full perception regarding their local reactions to be measured. The VAPI questionnaire is composed of 9 scores providing a comprehensive appraisal of acceptance of vaccination, with each score measuring a different aspect of subjects' perceptions. The complex structure of the VAPI highlights the multifaceted nature of subjects' perception regarding vaccination, that is different depending on whether it is assessed before injection, during injection or after injection. The VAPI questionnaire allows how subjects distinguish what they feel at these different assessment times, and how the vaccination impacts their level of willingness to be vaccinated again to be captured. In addition, the questionnaire was simultaneously developed in US English, French and German, and subsequently translated and culturally adapted into Belgian Dutch, UK English, Spanish and Italian, which will help in promoting its wider use in further international studies. Its self-administered nature will facilitate its use in large studies and any studies where there is a need to optimise the clinicians' involvement.

Our questionnaire was globally well accepted. It can be considered to be easily understandable, as more than 99% of the questionnaires from a large population sample (comprising adults and seniors) from multiple European countries with differing attitudes towards vaccination were more than 50% complete. A limitation might lie in the way subjects were recruited for interviews: via general practitioners in the US or via clinical trials in Germany and Switzerland. This may have introduced a bias in the information gathered from these interviews on three levels. Firstly, whereas European interviewees had received an extensive and detailed information sheet as part of the clinical trial informed consent process, US interviewees were recruited directly by their general practitioners and did not have access to the same level of information. Similarly, clinical trial subjects were required to complete a diary card therefore actively looked for certain reactions, whereas US interviewees did not. Altogether, this may have an impact on the coherence of the information when being pooled.

However the high stability of the structure and internal consistency validity of the multi-item dimensions were demonstrated in the different languages. Regarding the individual items, no data were available to test their reliability in these studies. Moreover, the test-retest reproducibility of the scores could not be evaluated in the framework of these clinical trials. The reliability (both internal consistency and reproducibility) of the VAPI remains to be further tested in future studies.

Of particular note are the very low scores (up to 1.58 on a 5-point Likert scale) and the high proportion of vaccinees answering most questions with the lowest possible score (i.e. 1), the most favourable perception of vaccination. This shows that, while these subjects reported ISR, they did not judge them to have negatively affected their quality of life or daily activities. As a further illustration of this, the willingness to be vaccinated the following year was not affected by the severity of reactions. This was true in all six countries considered. So far, the questionnaire has only been used in clinical trial volunteers. As these volunteers are likely to be less concerned by vaccination than the general population, a real test of the questionnaire would be its use in this latter population, i.e. one that includes those who strongly dislike vaccination.

Mean results for all dimensions and individual items were slightly lower among older adults, which is in line with studies showing that older adults report fewer and less severe reactions after influenza vaccination [39]. ISR severity correlated with the mean score of the multi-item "bother from ISR" dimension (milder reactions correlated with lower mean score), but the correlation between severity with the individual "bother" items was low. Together, these data highlight the ability of multi-item dimensions, but not individual items of a dimension, to discriminate between severity groups.

Conventional diary cards and the case report forms used in vaccine clinical trials typically describe adverse reactions in terms of quantitative variables (time to onset, duration, severity and outcome). The VAPI questionnaire could be used to provide additional qualitative information about the effect of the reactions on daily life, as well as on the overall perceptions of vaccination. Results could then be used to aid clinicians to inform subjects, not only about the possible reactions they might have following vaccination, but also the likely impact that such reactions will have.

## Conclusion

The self-administered VAPI questionnaire is a valid and reliable instrument for the assessment of the perceived impact of ISR on vaccinees' daily activities, the acceptability of these reactions, and the vaccinees' overall satisfaction with the vaccination.

## Competing interests

Catherine Chevat and Van Hung Nguyen are sanofi pasteur employees. Muriel Viala-Danten and Carla Dias-Barbosa are Mapi Values employees. This project was funded by sanofi pasteur and conducted by Mapi Values.

## Authors' contributions

CC was responsible for the study conception; participation in the development, psychometric validation and interpretation steps of the questionnaire. MVD was responsible for methodological directives and input for the questionnaire finalisation, validation and scoring analyses. CDB participated in questionnaire development. VHN was responsible for the study conception and interpretation steps of the questionnaire.
